# Monitoring and Managing Lifestyle Behaviors Using Wearable Activity Trackers: Mixed Methods Study of Views From the Huntington Disease Community

**DOI:** 10.2196/36870

**Published:** 2022-06-29

**Authors:** Philippa Morgan-Jones, Annabel Jones, Monica Busse, Laura Mills, Philip Pallmann, Cheney Drew, Astri Arnesen, Fiona Wood, Domino-HD Consortium

**Affiliations:** 1 School of Engineering Cardiff University Cardiff United Kingdom; 2 Division of Population Medicine Cardiff University Cardiff United Kingdom; 3 Centre for Trials Research Cardiff University Cardiff United Kingdom; 4 European Huntington Association Oslo Norway; 5 see Acknowledgments

**Keywords:** Huntington disease, activity tracker, perceptions, digital technologies, physical activity, qualitative research, survey

## Abstract

**Background:**

There are early indications that lifestyle behaviors, specifically physical activity and sleep, may be associated with the onset and progression of Huntington disease (HD). Wearable activity trackers offer an exciting opportunity to collect long-term activity data to further investigate the role of lifestyle, physical activity, and sleep in disease modification. Given how wearable devices rely on user acceptance and long-term adoption, it is important to understand users’ perspectives on how acceptable any device might be and how users might engage over the longer term.

**Objective:**

This study aimed to explore the perceptions, motivators, and potential barriers relating to the adoption of wearable activity trackers by people with HD for monitoring and managing their lifestyle and sleep. This information intended to guide the selection of wearable activity trackers for use in a longitudinal observational clinical study.

**Methods:**

We conducted a mixed methods study; this allowed us to draw on the potential strengths of both quantitative and qualitative methods. Opportunistic participant recruitment occurred at 4 Huntington’s Disease Association meetings, including 1 international meeting and 3 United Kingdom–based regional meetings. Individuals with HD, their family members, and carers were invited to complete a user acceptance questionnaire and participate in a focus group discussion. The questionnaire consisted of 35 items across 8 domains using a 0 to 4 Likert scale, along with some additional demographic questions. Average questionnaire responses were recorded as positive (score>2.5), negative (score<1.5), or neutral (score between 1.5 and 2.5) opinions for each domain. Differences owing to demographics were explored using the Kruskal-Wallis and Wilcoxon rank sum tests. Focus group discussions (conducted in English) were driven by a topic guide, a vignette scenario, and an item ranking exercise. The discussions were audio recorded and then analyzed using thematic analysis.

**Results:**

A total of 105 completed questionnaires were analyzed (47 people with HD and 58 family members or carers). All sections of the questionnaire produced median scores >2.5, indicating a tendency toward positive opinions on wearable activity trackers, such as the devices being advantageous, easy and enjoyable to use, and compatible with lifestyle and users being able to understand the information from trackers and willing to wear them. People with HD reported a more positive attitude toward wearable activity trackers than their family members or caregivers (*P*=.02). A total of 15 participants participated in 3 focus groups. Device compatibility and accuracy, data security, impact on relationships, and the ability to monitor and self-manage lifestyle behaviors have emerged as important considerations in device use and user preferences.

**Conclusions:**

Although wearable activity trackers were broadly recognized as acceptable for both monitoring and management, various aspects of device design and functionality must be considered to promote acceptance in this clinical cohort.

## Introduction

### Background

Huntington disease (HD) is a hereditary degenerative neurological disease that affects between 6 and 13 people per 100,000 in the general population [1]. The disease is characterized by the complex presentation of clinical symptoms involving motor, cognitive, and behavioral impairments [2]. People with HD experience a progressive decline in their quality of life and function over 15 to 20 years and premature death [2].

There are indications that lifestyle behaviors such as physical activity and sleep may be linked to the onset and progression of HD [3], with a systematic review indicating preliminary support for the benefits of exercise and physical activity in HD [4] and the European Huntington’s Disease Network producing a Physiotherapy Guidance Document for HD [5] supporting physical activity. Sleep disturbance is another feature of HD [6]; however, it is difficult to obtain objective measures of physical activity and sleep that are clinically relevant and valid. Previous studies have relied on subjective reporting from patients, which can be subject to recall bias, high levels of missing data, and can take substantial time to complete, leading to significant measurement bias in the results [7].

Wearable activity trackers may provide a suitable platform to objectively capture physical activity and sleep data. There has been a surge in the availability of wearable digital technology in the consumer market for measuring daily activities and lifestyle habits [8]. Short-term goal setting and instant feedback abilities make them efficacious motivational tools to promote health-related behaviors, and they are increasingly used to facilitate the management of some long-term conditions [9,10].

Consumer engagement with these devices is complex [11]. Although some people may hold a largely positive attitude toward digital wearable devices, others may regard them as invasive or intrusive. Adherence (ie, wearing the device) can be an issue, with some clinical research studies showing that the device was only worn for approximately 50% of the time over 2 days [12,13]. Concerns have also been raised regarding the validity and reliability of the recorded data [14]. Therefore, researchers must consider the balance between clinical validity and consumer compatibility when selecting an appropriate activity tracker device for research and clinical use.

### Objective

The Multi-Domain Lifestyle Targets for Improving Huntington Disease (DOMINO-HD) study is a multinational observational study that aims to explore the interplay between lifestyle and genetic factors and HD outcomes. The study hypothesizes that the prognosis of HD can be influenced by the use of digital technology to detect symptoms and allow modification of lifestyle factors. The aim of the DOMINO-HD substudy reported here is to gain an understanding of the views of individuals with HD and their families or caregivers on digital wearable activity tracker devices that will be used in the wider DOMINO-HD research program. It is vital to explore the experiences and preferences of patients with HD and their caregivers regarding wearable activity trackers to maximize downstream engagement with the technology in the DOMINO-HD study.

## Methods

### Study Design

A mixed methods approach was used by combining self- or proxy-completed questionnaires and focus group discussions. Using mixed methods research allowed us to integrate both types of data to seek a wider range of attitudes [15]. Our questionnaire data allowed us to assess the extent of support for activity trackers in relation to predetermined questions. Our focus group data allowed us to gain insights into participants’ explanations of their views, decision-making processes, and previous experiences. In designing our research, we drew on the Technology Acceptance Model, which examines how users come to accept and use a technology [16].

### Recruitment

Opportunistic recruitment took place from September to November 2019 at Huntington’s Disease Association meetings. These included the patient-centered 2019 European Huntington’s Association (EHA) meeting in Bucharest, Romania, and regional Huntington’s Disease Association meetings for patients and family members across the United Kingdom (Belfast, Northern Ireland; Cardiff, Wales; and Telford, England). Members of the DOMINO-HD research team attended these meetings, distributing promotional materials and inviting people to participate in both the questionnaire and focus group aspects of this study.

The inclusion criteria to participate in both the questionnaire and focus groups included being aged ≥18 years and having a genetically confirmed diagnosis of HD or being a family member or carer for someone who has HD. This facilitated the collation of opinions across a range of stakeholders within the HD community.

As no personal or sensitive data were requested within the questionnaire, potential participants were made aware that completion of the voluntary questionnaire would be regarded as providing their consent to participate in the study. Those participating in the focus groups were provided with an information sheet and asked to provide written informed consent before participating. As the focus groups were conducted during the conference, participants may have had only a few hours to consider whether they wished to participate, but the facilitator was mindful of explaining that they could withdraw if they wished. Furthermore, no sensitive topics or clinical information were discussed and shared during the focus groups.

### Questionnaire Design

Participants were asked to complete a questionnaire on their thoughts on wearable technology that can monitor lifestyle behaviors (Multimedia Appendix 1). More specifically, an adapted version of the questionnaire developed by Wu et al [17] was used; the questionnaire had originally been designed to explore consumers’ intention to accept a smartwatch, with a recognized potential to be extended to other wearable device studies. The modifications made were based on feedback from a Patient and Public Involvement workshop and related to the layout of the questionnaire (visualizing the Likert scale with colored smiley faces of varying happiness and reducing the number of questions per page). Slight alterations to the wording of section 1 of the Wu et al [17] questionnaire were made to reflect the potential benefits of using activity trackers within the HD community.

A total of 39 questions fell within eight subsections: relative advantage, ease of use, compatibility, result demonstrability, enjoyment, social influence, attitude, and behavioral intention (refer to Table 1 for each section descriptor). Each question within these subsections was measured using a 5-point Likert scale based on negative and positive anchors, ranging from 0=*strongly disagree* to 4=*strongly agree*. An additional option, *I don’t know*, was also available. Two additional questions were asked to identify the participants’ association with HD (ie, whether they had a genetically confirmed diagnosis of HD or were a family member or carer of someone with HD) and to determine their age group (≤24 years, 25-34 years, 35-44 years, 45-54 years, and ≥55 years).

**Table 1:** Questionnaire domains, descriptors, and example statements.

Questionnaire domain	Section descriptor	Example statement
1. Relative advantage	Whether wearable activity trackers are perceived to be beneficial to those with HD^a^	“The activity tracker would help me to monitor my physical activity and sleep.”
2. Ease of use	Whether people with HD would find wearable activity trackers easy to use	“I believe that the activity tracker would be easy to use.”
3. Compatibility	Whether activity trackers would be compatible with people who have HD	“An activity tracker is something that I can see fitting my current habits.”
4. Result demonstrability	Whether people with HD would be able to understand the information from a wearable activity tracker and be able to explain it to others	“Observing how I do things differently before and after using an activity tracker would be easy for me.”
5. Enjoyment	Whether people with HD would enjoy using a wearable activity tracker	“Using an activity tracker would be an ideal recreation”
6. Social influence	Whether people with HD would find wearable activity trackers helpful in raising their social status and whether they value the opinion of others on whether they use such a device	“Anyone who uses a fitness tracker would have higher social status within my social circle.”
7. Attitude	Whether people with HD have a positive attitude toward wearing a wearable activity tracker	“Using an activity tracker would be a positive decision.”
8. Behavioral intention	Whether people with HD would be willing to use a wearable activity tracker	“I would be willing to use an activity tracker.”

^a^HD: Huntington disease.

Participants were able to complete the questionnaire on paper or electronically (via the Bristol Online Surveys platform), which took approximately 10 to 15 minutes to complete. The participants were alerted to this study via a flyer in their delegate packs, which contained a QR code with a link to the questionnaire. A member of the research team was always present at a stand at the conference, allowing delegates the opportunity to complete the questionnaire on an iPad. The paper versions were also handed out and then returned to the researchers at the stand. Given that the European participants attended the EHA Bucharest event, the questionnaire was made available in English, Polish, German, and Spanish, with translations from English being made by DOMINO-HD partners.

### Focus Groups

Each focus group was conducted in English, involved 4 to 6 participants, and was facilitated by a researcher trained and experienced in the method and research related to patients with HD. In addition, an additional observer from the research team was present to support the group and make notes. Owing to the relatively narrow aims of the focus groups, we anticipated that we would need to conduct 2 or 3 focus groups to achieve data saturation. They lasted approximately 1 hour (although timings were flexible to the needs and wishes of the participants involved in each group) and were audio recorded using an encrypted audio recorder. They were conducted in a private room during the conference. A semistructured topic guide, developed to explore patients’ attitudes toward wearable devices in osteoarthritis [18], was modified for relevance and used to guide the topics (Multimedia Appendix 2). In addition, short focusing activities were conducted, including a group ranking in which participants were asked to collectively rank in order of importance the features of activity trackers. The main purpose of the ranking exercise was to facilitate discussion without interpreting it quantitatively. Finally, participants were asked to discuss a vignette scenario around wearable technology (Multimedia Appendix 3). The list of items to be ranked and the vignette were developed by the research team with input from the DOMINO-HD Patient and Public Involvement group. The research team was from a range of backgrounds, including physiotherapy (research and clinical), engineering, medicine, and sociology. Our multidisciplinary background encouraged us to explain and keep checking our various assumptions and biases during data collection and analysis.

### Data Analysis

All questionnaire responses were entered into the digital version of the questionnaire, where responses were coded and exported using Microsoft Excel. The data were then imported into Spyder (Python 3.8; Python Software Foundation) for further analysis using Pandas [19] and NumPy [20] Python packages. Missing and *I don’t know* responses were recoded as *not a number* before the responses to questions within each section of the questionnaire were averaged for each participant. Participants were required to respond to a minimum of 1 question within a given section of the questionnaire to be included in the analysis for that section.

For each questionnaire subsection, responders were divided into those with a positive opinion (ie, producing a mean section score of >2.5), neutral opinion (ie, producing a mean section score of 1.5-2.5), or negative opinion (ie, producing a mean section score of <1.5) and reported as a percentage of the total number of responders for each subsection. The median cohort response for each subsection was also reported alongside the IQR and similarly interpreted in relation to positive, neutral, or negative opinions. A more elaborate quantitative analysis of the association between responses from each questionnaire subsection was not possible as part of this study because of the nature of the responses collated (limited variance across participants and/or questionnaire subsections).

A Wilcoxon rank sum test was performed to determine whether questionnaire responses differed between those with a confirmed diagnosis of HD and those who were family members or carers for someone with HD (*a priori* level of significance set to *P*=.05, with *P* value Holm correction performed for multiple comparisons). A Kruskal-Wallis test was performed to determine whether questionnaire responses differed between the 5 age categories (*a priori* level of significance was set to *P*=.05, with *P* value Holm correction performed for multiple comparisons).

All focus groups were audio recorded and transcribed verbatim by a professional transcription agency. Transcriptions were read and checked for errors by a researcher who was present in all focus groups, and the primary identifiers were deidentified. An exploratory thematic analysis [21] was performed by a single researcher using the NVivo 12 software (QSR International). After conducting 2 focus groups, the team reflected on the data saturation and determined that a third focus group would be beneficial. Following the additional analysis of these data, the team deemed that we had reached a point of data saturation. The topic themes allocated to nodes were mutually agreed upon across the broader research team and refined during the coding process as topics were unearthed. A third of the data were double coded by another researcher, and then coding decisions were discussed to encourage a more explicit engagement with the data and check coding consistency [22].

The themes identified through the focus group thematic analysis were used to provide context for the questionnaire responses and explore the underpinning opinions of the HD community toward wearable activity trackers.

### Ethics Approval

Ethics approval was obtained from the Cardiff University School of Medicine Ethics Committee (ref 19/71, September 19, 2019).

## Results

### Questionnaire Results

A total of 114 participants completed the study questionnaire, of which 9 (7.8%) were removed from the data set owing to incomplete data (n=1) or failing to fall within the demographics of interest (having a genetically confirmed diagnosis of HD or being a family member or carer of someone with HD [n=8]). This left a questionnaire cohort size of 105 with recorded demographics, as described in Table 2.

The participants were overwhelmingly positive regarding the use of wearable activity trackers (Table 3). All sections of the questionnaire produced a median cohort response >2.5, representing a tendency for positive opinions toward the use of wearable activity trackers (such as devices being advantageous, easy and enjoyable to use, and compatible with one’s lifestyle and users being able to understand the information from wearable activity trackers and willing to wear them).

The only differences observed were in the behavioral intention questionnaire response score because of age (Table 4), with the oldest age group (≥55 years) having a lower median response score than the two youngest age categories (≤24 years and 25-34 years). However, pairwise comparisons were not statistically different when *P* values were adjusted for multiple comparisons (adjusted *P*>.05). Responders who had a genetic diagnosis of HD were found to have a significantly more positive median response for the ease of use, enjoyment, attitude, and behavioral intention sections when compared with those who cared for or were a family member of someone with HD. However, this only remained statistically significant for the attitude section, following the Holm correction procedure (adjusted *P*<.05).

**Table 2:** Questionnaire participant demographics (N=105).

		Participants, n (%)
**Age group (years)**		
	≤24	7 (6.7)
	25-34	18 (17.1)
	35-44	23 (21.9)
	45-54	20 (19)
	≥55	35 (33.3)
	Not specified	2 (1.9)
**Association with HD^a^**		
	Having a genetically confirmed diagnosis of HD	47 (44.8)
	Being a family member or carer for a person with HD	58 (55.2)
**Language of questionnaire completion**		
	English	93 (88.6)
	Spanish	3 (2.9)
	German	5 (4.8)
	Polish	4 (3.8)

^a^HD: Huntington disease.

**Table 3:** The percentage of median positive, neutral, and negative responses along with the total number of responders for each questionnaire section^a^.

Questionnaire section	Positive responses^b^, %	Neutral responses^c^, %	Negative responses^d^, %	Cohort response, median (IQR; range)	Total number of responders (n)
Relative advantage	92.23	4.85	2.91	3.6 (3.0-4.0; 0-4)	103
Ease of use	81.55	14.56	3.88	3.4 (2.8-4.0; 0-4)	103
Compatibility	65.69	23.53	10.78	3.3 (2.3-4.0; 0-4)	102
Result demonstrability	78.43	17.65	3.92	3.0 (2.8-4.0; 0-4)	102
Enjoyment	60.61	27.27	12.12	3.0 (2.0-4.0; 0-4)	99
Social influence	53.92	34.31	11.76	2.6 (2.0-4.0; 0-4)	102
Attitude	74.26	18.81	6.93	3.2 (2.5-4.0; 0-4)	101
Behavioral intention	88.35	7.77	3.88	3.7 (3.0-4.0; 0-4)	103

^a^Participants were asked to rate on a scale of 0 (strongly disagree) to 4 (strongly agree).

^b^Scores>2.5.

^c^Scores between 1.5 and 2.5.

^d^Scores<1.5.

**Table 4:** Median and IQR for cohort-level questionnaire response scores and results when testing for differences in questionnaire response based on age categories (Kruskal-Wallis test) and whether they were a carer or patient (Wilcoxon rank sum test).

Questionnaire section	Kruskal-Wallis results			Wilcoxon rank sum results		
	Test statistic	*P* value	Adjusted *P* value	Test statistic	*P* value	Adjusted *P* value
Relative advantage	6.195	.19	.79	1.996	.046	.23
Ease of use	6.486	.17	.79	2.343	.02^a^	.12
Compatibility	6.61	.16	.79	1.07	.29	.57
Result demonstrability	7.87	.10	.67	1.558	.12	.36
Enjoyment	5.864	.21	.79	1.986	.047^a^	.23
Social influence	5.234	.26	.79	0.802	.42	.57
Attitude	7.903	.10	.67	3.073	.002^a^	.02^a^
Behavioral intention	9.773	.04^a^	.35	2.602	.009^a^	.07

^a^Statistically significant *P* values (at the 5% level).

### Focus Group Results

#### Overview

A total of three focus groups were conducted: 2 in Bucharest at the EHA patient conference (involving 5 participants in the first focus group and 6 participants in the second), and 1 in Cardiff at the patient HD meeting (involving 4 participants). The demographics of the focus group are presented in Table 5.

The results of the group ranking exercise undertaken during the focus groups are shown in Table 6. Although there were obvious differences in how each focus group ranked what was important to them, the features that appeared to be most important were accuracy, comfort, and ease of use. Appearance and ability to use the watch in other aspects of their lives were the least important.

A total of 4 overarching themes were developed to describe the acceptability of wearable activity trackers to people with HD. These included the accessibility and compatibility of a device, its impact on a person’s relationships, whether it can be used effectively for self-management and monitoring of lifestyle behaviors, and the security of the data being collected. We discuss each of these themes in turn with illustrative extracts from the focus groups, although we have withheld participant characteristics from the quote to prevent deductive disclosure because of concerns about the HD community being relatively small.

**Table 5:** Focus group participant demographics (N=15).

		Cohort sample size, n (%)
**Gender**		
	Male	5 (33)
	Female	10 (67)
**Experience with wearable devices**		
	Yes	5 (33)
	No	10 (37)
**Genetic status**		
	Having a genetically confirmed diagnosis of HD^a^	5 (33)
	Not disclosed	10 (67)
**Country of residence^b^**		
	United Kingdom	7 (46)
	Other European country	4 (27)
	Other	4 (27)

^a^HD: Huntington disease.

^b^Categories have been collapsed for participants’ stated country of residence owing to small numbers.

**Table 6:** Results of the ranking exercise in which participants were asked to rank the most important factors that would determine their engagement with a wearable device.

Rank	Group 1	Group 2	Group 3
1	Ease of use	Accuracy	Accuracy
2	Getting feedback from the device	Cost	Comfort
3	Comfort	Comfort	Ease of use
4	Keeping data safe and secure	Battery life	Where the tracker is located on the body
5	Accuracy	Keeping data safe and secure	Keeping data safe and secure
6	Battery life	Obtaining feedback from the device	Obtaining feedback from the device
7	Where the tracker is located on the body	Ease of use	Cost
8	Cost	Where the tracker is located on the body	Battery life
9	Being able to use the watch for things not related to the study	Being able to use the watch for things not related to the study	Appearance
10	Appearance	Appearance	Being able to use the watch for things not related to the study

#### Theme 1: Accessibility and Compatibility

Focus group participants acknowledged that wearable devices must be easy to use for people with HD to engage with them. This was deemed particularly important as this cohort was thought to have a wide-ranging level of digital technology experience. However, the participants were clear that they did not want an easy-to-use device at the expense of limited functionality. Rather, they were clear that they valued the special features of many commercially available activity watches:

When you hit 10,000 steps and you’ve got it on your wrist there’s these like fireworks go off...and it vibrates and whatever and you know, you’re wonderful and then on the actual app itself, like on the phone, erm, that it’s synced to, it’s, like I thought it was really clever.

All participants agreed that battery life was an important factor when considering if a device was easy to use. It was acknowledged that although charging a device was a necessity, a short battery life was not synonymous with ease of use. Those who already owned a wearable device noted how often and for how long charging was required:

The charge takes about two hours, this, I, I, I done a lot of research and for the money, erm, and for all round what it does, this ticks all the boxes for me.

It was also felt that battery life diminished over time, and devices often failed to reach the battery lengths advertised:

You know the Fitbits say they’re supposed to last five days, they don’t last five days, no way they last five days. I charge mine every night.

Concerns were raised as to whether people with HD, particularly as the disease progresses, would be able to remember to remove a device, charge it, and put it back on. This process needed to be simplified and as infrequent as possible. A device that is waterproof and, therefore, reduces the need to take off and put back on again was similarly deemed beneficial. It was also recognized that a simple watch strap would be required so that those with progressive motor symptoms would be able to wear and remove the device. One participant mentioned using different types of straps with their current device, including a magnetic strap that easily clicks together.

The preferred location for a wearable activity tracker was the wrist; however, interest was also shown in devices that could be worn under clothes so that they could be worn more discreetly. Comfort was believed to be a key factor in device adoption, with participants wanting a device that was as unobtrusive as possible and not bulky or large. However, it was recognized that if there was a need and benefit from using a device, then appearance was not a critical factor in device adoption:

If you have the need, it looks like whatever it looks like.

Several participants displayed a reluctance to wearing a device at night, owing to continuous monitoring feeling too invasive and onerous or simply because they do not wear a watch to bed. Although others did accept wearing a device at night for the purpose of research, the overall consensus suggested that nocturnal monitoring with a wearable activity tracker is not compatible in a real-world context:

Ultimately to be a part of somebody’s medical care then it has to be as less invasive as possible, okay? Because I don’t sleep with watches, I’m not going to enjoy sleeping with watches.

Cost was believed to be a limiting factor; although some were prepared to pay for a high-end device if it met their requirements, others noted that some HD families have financial constraints that must be considered.

Participants believed that wearable activity trackers are generally considered compatible with the lifestyle and daily routine of HD families and highlighted how the cohort often uses such technologies already. It was recognized that the adoption of a given device within this clinical cohort required iOS and Android operating system compatibility. Wearable activity trackers were deemed particularly suited to younger people, as they were seen to fit well with the global trend of sharing information via social media. Participants felt that remote health monitoring at home had considerable future potential and that similar technologies were needed for patients with HD. There was a view that, where possible, the person with HD should be encouraged to engage with the wearable device themselves to promote autonomy and engagement.

#### Theme 2: Impact on Relationships

The participants discussed the difficulties of striking a balance between using the device as a helpful tool and ensuring that it did not become socially or emotionally consuming. Dependence was felt to lead to unnecessary stress when trying to meet health-related targets:

It does seem to me that addiction is a big issue. I mean I’ve certainly read that that is the case, like some people they do get quite obsessed with targets and, and you know, if they haven’t done it, they get quite worked up and stressed...

Feedback, although essential for motivation and engagement, was also thought to contribute to frustration. Tracking and monitoring may prove overwhelming for some patients, particularly those with psychiatric conditions, as one participant illustrated when discussing their experience in attempting to *track* and help their sibling with prompts:

He said, oh all of these numbers, you know, they’re driving me crazy, you know, and it was really, it was really freaking him out.

Monitoring sleep difficulties was also a source of frustration for some participants:

Now the sleep one, even though it tells me you’re not sleeping, it doesn’t help me, it doesn’t tell me what I can do...

It’s inventing more stress as a consequence.

Gaining pleasure from a device was considered possible. For a patient with HD, gaining a sense of autonomy over how they manage their disease could improve their outlook and subsequent relationships with others:

If the individual feels that by doing this they’re taking more control of their situation that’s got to be good for the outlook and the interaction with people around them.

However, it was acknowledged that if a carer or family member was to be heavily involved in the use of the device, a patient may lose their sense of autonomy and subsequently be less likely to engage. It may become an irritant and potentially strain the carer-patient relationship:

I can imagine, like a carer or a family member that’s trying to promote it for the person to do it, it might not be as successful if the person wants to do it themselves and take ownership of it I suppose.

Nearly all participants thought that wearable devices would improve their interactions with medical professionals. Clinicians could analyze longitudinal data produced by the device and modify treatment plans accordingly. However, some have suggested that these devices may make some clinicians feel that their clinical experience and judgment are being challenged:

Some doctors would definitely take the view that you’re trying to do their job for them and not like that. Others would welcome the increased data that they can make judgements on, I’ve run into both.

#### Theme 3: Self-management

The participants suggested that wearable activity trackers opened up the possibility of new ways for clinicians, researchers, and patients to diagnose, monitor, and manage HD. The need to improve how patients with HD interact with their health professionals was noted, along with the advantages of generating longitudinal data to assist in clinical decision-making rather than relying on how a patient presents on a particular day in the clinic:

It gives data that is continuous, so now you have a sense over time, all the time, not just when the person goes to hospital or goes to clinic.

Those who had experience of using wearable activity trackers described them as being an integral component of a healthy lifestyle, giving them motivation and encouragement to reach their goals and a way of recording success:

It just helps me track my health and fitness goals, really. It keeps me on track and focussed. Erm, yeah, keeps me focussed I think is the main advantage for me.

There was a strong desire to receive feedback from a wearable activity tracker to increase personal motivation, allowing the visualization of goals:

I will make that extra effort to hit the target if I’m near it.

However, the participants recognized that not hitting short-term targets may lead to disappointment and subsequent demotivation. For someone with HD who is seeing a decline in function and potentially not meeting their activity targets, this decline in function may be psychologically damaging:

If this is tracking disease progression then if Peter [person in the vignette] was to see a significant change in his disease they may, actually may make him more depressed or more concerned and actually be less helpful to him.

Although many benefits of using wearable activity trackers were noted, there was a strong feeling that these will only be realized if wearable technologies can record measurements with a reasonable level of accuracy (with mention of an 85% accuracy threshold considered as acceptable):

If it’s not accurate to the degree that it needs to be then there’s no point in doing any of this.

Let’s face it, I mean if it’s not accurate it’s a complete waste of time.

The participants also believed that some medical professionals were not very receptive to patients producing their own data. Participants believed that getting clinicians involved in the process of deciding what to measure and how to feed back to health professionals could improve their acceptance of the technology.

#### Theme 4: Data Security

Concerns about data security were also raised. It was felt that the misuse of personal data by third parties could lead to potential discrimination and that people with neuropsychiatric disease features may be obsessed with data security. Despite this, people were willing to still engage with a device and share personal information if there was perceived benefit and little risk:

Keeping your data safe and secure though would be very important for some people I’d imagine.

## Discussion

The long-term adoption of wearable activity trackers requires users to accept technology in their daily lives. This study sought to understand the potential views and opinions of the HD community toward wearable activity trackers to guide the selection of a wearable activity tracker for use in a longitudinal observational clinical study.

### Principal Findings

The responses to wearable devices in the context of HD were largely positive in both the questionnaires and focus groups. Activity trackers need to be accurate, have a useful purpose, be easy and enjoyable to use, and be compatible with the wearer’s lifestyle. It was also considered important that this patient group would be able to understand the information received from the tracker. Older participants were less likely to indicate that they would be willing to use a wearable device than younger participants (behavioral intentions). Our focus group data indicated that engagement with the device was facilitated by accuracy, ease of use, comfort, usable feedback, and reliable battery life. The questionnaire data showed that the domain of social influence, or how the activity tracker might affect how other people view the user, was less important. Again, this was paralleled in the focus group data, where the appearance of the device was deemed to be less important in the ranking exercise. Concerns were also raised regarding the suitability of using a wearable device for some patients with HD, particularly those with significant neuropsychiatric symptoms. Reassurances regarding data security also need to be considered.

### Findings in the Context of Other Literature

The Technology Acceptancy Model suggests that when users are presented with a new technology, the main factors that influence their intention to use it are perceived usefulness and perceived ease of use [16]. Our data indicate that activity trackers need to have a useful purpose. Usefulness was associated with accuracy in attaining benefits. However, high thresholds of accuracy, suggested by one of our focus group participants, are unrealistic, particularly because of the uncertainty of the accuracy and reliability of data from commercial devices [23]. The expectation of having an accurate wearable activity tracker is consistent with several other studies [24]. Although no participants in this study directly questioned the accuracy of wearable activity trackers, the algorithms embedded within devices are typically developed using the movement patterns of healthy people, which may lead to higher data inaccuracies when applied to people with HD, who often present with motor symptoms involving their upper limbs and pathological gait characteristics. A recent review of wearable activity tracker research [25] found a substantial increase in the literature focusing on wearable activity trackers in recent years, with the most common research theme focusing on concerns about the reliability, accuracy, and validity of the technology itself.

Research has also suggested that wearable devices worn on the wrist have a greater error rate than those worn on other parts of the body [26]. Despite this, the immediate feedback provided by a digital screen on a wrist-watch device, which is essential for sustained user engagement, indicates that it is the preferred location for many users [26]. Accuracy may not need to be perfect, but it is important and must be sufficient to produce reliable clinical data. Researchers will also need to know more about the inaccuracies of measurement to appreciate the minimal clinically relevant differences between populations.

Acceptability is also linked to the ease of use. A device that was small, discrete, and comfortable was preferred. Most participants were not supportive of a cumbersome device that could lead to patient identification and consequent discrimination, which were concerns shared by other neurodegenerative populations [27,28].

Despite numerous potential benefits, concerns have been expressed regarding the relationship with a device that measures several behaviors in this specific patient population. Most research on wearable devices is primarily focused on healthy populations, and research on clinically specific populations is relatively scarce [18]. Therefore, there is a need to consider the risk of harm in the HD population. Disease features such as depression, anxiety, and cognitive impairment can influence social relationships, including engagement with a device [29,30]. Some users may become dependent on wearable technology and place a disproportionate value on the data produced, with consequences on mood and further engagement [31]. Lack of confidence in technology has also been cited as a barrier to engagement in other neurodegenerative diseases, with some patients expressing stress at the thought of wearing such a device [32]. Others may find it intrusive and uncomfortable [14]. Although compliance may not be an issue, welfare concerns can arise. Therefore, researchers and clinicians must assess the suitability of participants using a device on an individual basis to prevent negative psychological consequences. Devices that do not offer user interaction or feedback may prevent this. However, this study suggests that devices with added functionality can increase patient adherence and engagement. Therefore, it is difficult to balance protecting patient vulnerabilities and maximizing user engagement and adherence by providing feedback.

The reluctance to wear a device at night is also shared by other populations, although the importance of continuous monitoring to obtain high-quality sleep data has been recognized [18,33]. Concerns raised about having to remember to physically charge a wearable device were also shared by patients with Parkinson disease, who cited difficulties in charging a device as a barrier to user engagement [27], partly owing to limited motor control and the dexterity needed to remove the device. Therefore, the design should aim to preserve the battery life and minimize charging requirements.

Concerns regarding data privacy were also raised in this study. These concerns are echoed in the literature [24,25,33]. Although patients may be open to sharing their personal information for the purpose of clinical benefit, there is a risk of data misuse. Therefore, it is vital to ensure that patient information is protected and that both researchers and industries demonstrate transparency regarding data use [33]. However, the literature suggests an overall risk-benefit consideration that favors engagement with wearable devices [34,35].

Participants’ perceived motivation and engagement in health-related behaviors because of wearable device use are shared by other research focusing on exercise and rehabilitation in chronic diseases [36,37]. Behavior change techniques instilled by wearable technology and driven by feedback, such as short-term goal setting, prompts, and reward systems, are key features that drive health surveillance [38]. In the context of long-term conditions, wearable technology may overcome limitations in monitoring patient-reported outcomes and encourage greater patient-clinician cooperation [37]. Consistent and reliable measurements provide an opportunity to identify and treat those with active disease and those who are at risk [38]. This has already been recognized by other neurodegenerative populations, and the use of wearable technology has already been introduced in the management of Parkinson disease [28,39]. The clinical application of wearable devices in a real-world context in HD remains limited, although this study suggests that they would be well received.

### Limitations

Although these findings were elicited in the context of HD, we believe that they are applicable to other long-term conditions, particularly those that can be influenced by health-related behaviors. We note that the questionnaire for the survey was not validated for the HD population or any other neurodegenerative population. However, we received feedback from our Patient and Public Involvement group about its relevance and acceptability. Our questionnaires were available only in 4 languages and were not back translated. However, the vast majority of the participants completed the questionnaire in English. Opportunistic sampling from HD meetings was deemed the most convenient recruitment method for this study. A purposeful sample strategy, which sought a balance of gender, age, and nationality of our participants, might have helped the representativeness of the sample but was considered impractical when recruiting at site. Therefore, our findings may not be generalizable to a broader cohort of patients with HD and their caregivers. We opted to collect only brief sociodemographic data on age group and whether the participant was a patient with HD or a caregiver, as we thought these might be the most important differences. However, we acknowledge that we were not able to report participants’ views in relation to gender, socioeconomic status, or educational background. In addition, focus group research can result in social desirability bias, leading to overestimation of a positive response [40], but this may have been rebalanced by our mixed methods design, where participants were self- or proxy-completing the questionnaire and thus had limited and anonymous contact with the research team. Functionality issues, fatigue, and cognitive impairment may also have prevented members of the HD population from participating in our research [30,41,42]. Nonetheless, there was flexibility in our design to allow people to choose to participate in either our focus groups or to complete our questionnaire.

### Conclusions

Our participants demonstrated a positive attitude toward the application of wearable activity tracker technology. The results of this study guided the design features of the chosen wearable device to be included in the DOMINO-HD study. The selection of and appropriate modifications to a wearable device should maximize user engagement and adherence for home monitoring throughout the observational study period.

As our research population was relatively specific, we recommend that other studies test the acceptability of wearable activity trackers in populations with other chronic conditions. On the basis of the findings of this study, further investigation into an acceptable device measurement schedule and a more detailed investigation into the acceptability of sleep monitoring are also recommended. This study also informed our choice of device that matches patient preferences for the DOMINO-HD longitudinal study, but there is a need to validate that device and measure any inaccuracies to enable a confident estimation of what the minimal detectable change in lifestyle behavior might be.

## Appendix

Multimedia Appendix 1 Questionnaire.

Multimedia Appendix 2 Focus group topic guide.

Multimedia Appendix 3 Vignette for focus group.

## Abbreviations

DOMINO-HD: Multi-Domain Lifestyle Targets for Improving Huntington Disease
EHA: European Huntington’s Association
HD: Huntington disease
