# Supplementary material for: Monitoring and Managing Lifestyle Behaviors Using Wearable Activity Trackers: Mixed Methods Study of Views From the Huntington Disease Community
Source: JMIR Form Res. 2022 Jun 29;6(6):e36870. doi: 10.2196/36870 (PMC9280464; doi:10.2196/36870)
Supplement: Multimedia Appendix 1 [file formative_v6i6e36870_app1.pdf]

Centre for Trials Research
Canolfan Ymchwil Treialon

PARTICIPANT INFORMATION

DOMINOHD

TITLE

How do people with Huntington’s Disease and their family members feel about using digital devices to monitor and manage their lifestyle?

WHAT IS THE PURPOSE OF THIS STUDY?

The DOMINO-HD study will explore how lifestyle factors such as diet, physical activity and sleep may influence disease status. We are asking people with Huntington's disease (HD) to use wearable activity trackers (such as FitBit) at home for continuous registration of sleep and activity. We plan to use this information to develop lifestyle interventions for HD. As part of preparing for this study, we want to understand how HD family members feel about personal use of digital wearables. This survey has been developed to help us understand how best to use such devices within DOMINO-HD.

WHO CAN TAKE PART?

You can take part if you are <u>over 18 years of age</u> and are one of the following:

- Have a genetically confirmed diagnosis of HD.
- Are a family member or care for someone who has HD

WHAT WILL I HAVE TO DO IF I TAKE PART?

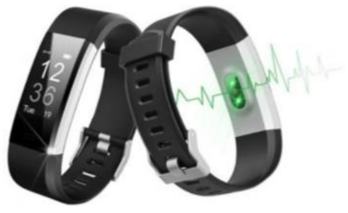

If you decide to take part, you will be asked to complete a questionnaire asking you what you think about using activity trackers (such as FitBit, Apple Watch or Garmin) in your daily life. We would expect it to normally take less than 15 minutes to complete this questionnaire and members of the DOMINO-HD team would be happy to help you.

HOW DO I ANSWER THE QUESTIONS IN THE QUESTIONNAIRE?

Please put a cross in the box under the face that best represents how you feel about each question if you have HD, **<u>or how you think your family member / person you care for who has HD would feel given your experience if you do not have HD</u>**.

Example question: Wearing a watch helps me keep track of the time.

Strongly Disagree	Disagree	Neutral	Agree	Strongly Agree	I don’t know.

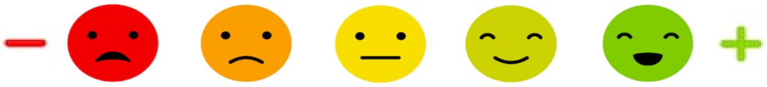

Strongly Disagree	Disagree	Neutral	Agree	Strongly Agree	I don’t know.
				X	

WILL MY DATA BE PROTECTED?

If you decide to complete the questionnaire we will assume that you have given us permission to use the data collected. Cardiff University will be responsible for storing all data collected in a secure and safe way. We will not record any personal information about you that would identify you so it will not be possible to identify you in any results that we may publish from this research.

CONSENT STATEMENT

DOMINOHD

TITLE

How do people with Huntington's Disease and their family members feel about using digital devices to monitor and manage their lifestyle?

By answering the questions within the questionnaire, you are:

1. Confirming that you understand what the study is about and what is being asked of people taking part in the study.

2. Confirming that you understand that participation is voluntary and that you are free to withdraw at any time without giving any reason.

3. Providing your consent to take part in the above study.

Please put a cross in the box under the face that best represents how you feel about each question

SECTION 1: Relative advantage

1. The activity tracker would become a helpful device for me.

– + I don't know.

2. The activity tracker would help me to monitor my physical activity and sleep

– + I don't know.

3. The activity tracker would provide my doctors with useful information.

– + I don't know.

4. The activity tracker would possess more advantages than disadvantages.

– + I don't know.

5. The activity tracker would help to motivate me to exercise more.

– + I don't know.

Please put a cross in the box under the face that best represents how you feel about each question

SECTION 2: Ease of Use

1. I believe that the activity tracker would be easy to use.

– + I don't know.

2. I believe that I could use the activity tracker flexibly (as I want to).

– + I don't know.

3. Learning to use the activity tracker would not be a challenge.

– + I don't know.

4. Using an activity tracker would not confuse me.

– + I don't know.

5. Becoming a skillful user of an activity tracker would be easy.

– + I don't know.

DOMINOHD

Please put a cross in the box under the face that best represents how you feel about each question

SECTION 3: Compatibility

1. I like the idea of using an activity tracker; it suits my lifestyle choices

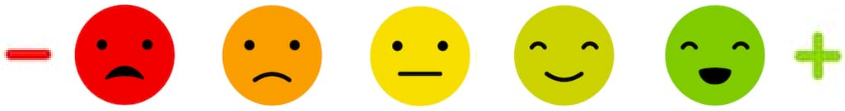

I don't know.

2. An activity tracker is something that I can see fitting my current habits

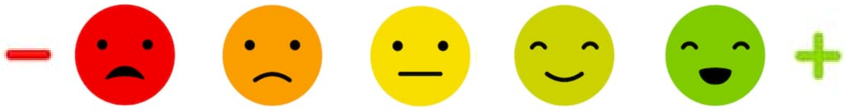

I don't know.

3. Using an activity tracker would match my lifestyle.

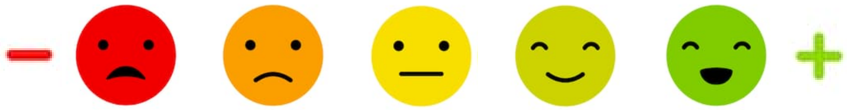

I don't know.

4. Using an activity tracker would fit with every aspect of my daily life.

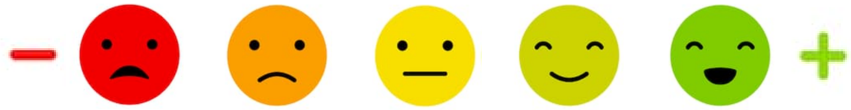

I don't know.

Please put a cross in the box under the face that best represents how you feel about each question

SECTION 4: Result Demonstrability

1. Observing how I do things differently before and after using an activity tracker would be easy for me.

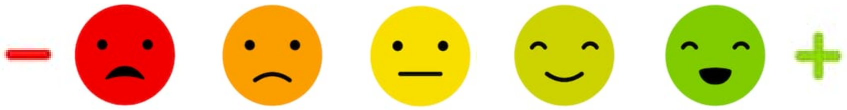

I don't know.

2. Expressing the results of using an activity tracker to other people would be easy.

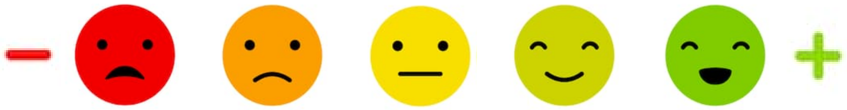

I don't know.

3. Expressing the benefits of using an activity tracker to other people would be easy.

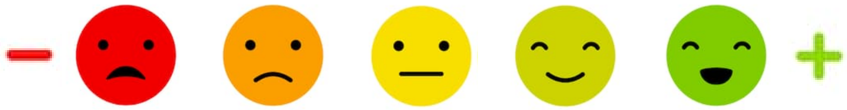

I don't know.

4. Expressing the importance of using an activity tracker to other people would be easy.

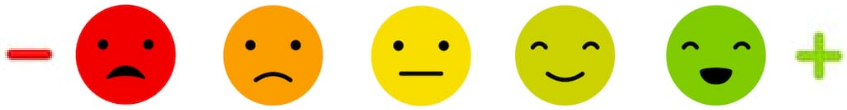

I don't know.

Please put a cross in the box under the face that best represents how you feel about each question

SECTION 5: Enjoyment

1. The process of using (interacting) with an activity tracker would make me feel happy.

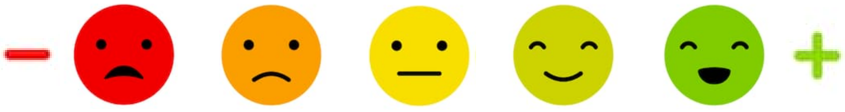

I don’t know.

2. Using an activity tracker would make me feel enjoyment.

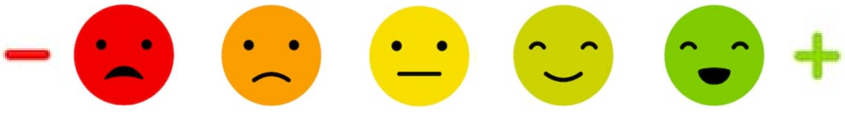

I don’t know.

3. Using an activity tracker would be an ideal recreation.

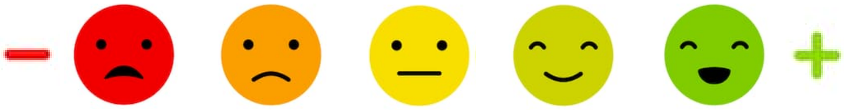

I don’t know.

4. Using an activity tracker would make me feel relaxed.

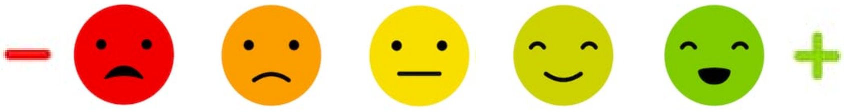

I don’t know.

DOMINOHD

Please put a cross in the box under the face that best represents how you feel about each question

SECTION 6: Social Influence

1. Using an activity tracker would help me to raise my social status.

I don't know.

2. Anyone who uses a fitness tracker would have higher social status within my social circle.

I don't know.

3. I value the opinion of my carers and health professionals as to whether I should use an activity tracker.

I don't know.

4. . I value the opinion of family and friends as to whether I should use an activity tracker.

I don't know.

5. People in my social circle would not object to my use of an activity tracker.

I don't know.

Please put a cross in the box under the face that best represents how you feel about each question

SECTION 7: Attitude

1. Using an activity tracker would be a positive decision.

– ☐ ☐ ☐ ☐ ☐ + I don't know. ☐

2. Using an activity tracker would be a smart decision to make.

– ☐ ☐ ☐ ☐ ☐ + I don't know. ☐

3. I have a positive impression toward using an activity tracker for work.

– ☐ ☐ ☐ ☐ ☐ + I don't know. ☐

4. I would feel excited to purchase an activity tracker.

– ☐ ☐ ☐ ☐ ☐ + I don't know. ☐

5. I would be happy to use an activity tracker.

– ☐ ☐ ☐ ☐ ☐ + I don't know. ☐

DOMINOHD

Please put a cross in the box under the face that best represents how you feel about each question

SECTION 8: Behavioural Intention

1. I would be willing to use an activity tracker.

– ☐ ☐ ☐ ☐ ☐ + **I don't know.** ☐

2. I would be willing to use an activity tracker if I possess one.

– ☐ ☐ ☐ ☐ ☐ + **I don't know.** ☐

3. I would be willing to use the information provided by an activity tracker to manage my lifestyle (e.g. perform daily exercises or monitor sleep).

– ☐ ☐ ☐ ☐ ☐ + **I don't know.** ☐

About Me (Please indicate the option that best describes your involvement in HD:

I have a genetically confirmed diagnosis of HD ☐

I am a HD family member or care for a person with HD ☐

Other: (Please give details)

____

____

Please tick the age category that you, or the person you have completed this for, falls within:

24 years or under	25 – 34 years	35 – 44 years	45 – 54 years	55 years or older
☐	☐	☐	☐	☐

<u>Thank you for your time, you have completed the questionnaire</u>
