# Supplementary material for: Monitoring and Managing Lifestyle Behaviors Using Wearable Activity Trackers: Mixed Methods Study of Views From the Huntington Disease Community
Source: JMIR Form Res. 2022 Jun 29;6(6):e36870. doi: 10.2196/36870 (PMC9280464; doi:10.2196/36870)
Supplement: Multimedia Appendix 2 [file formative_v6i6e36870_app2.docx]

### Supplementary Appendix 2. Focus group topic guide

1. Introduction

- Moderators and participants introduce themselves
- Clarification on the format of the focus group and aim
- Assurance of confidentiality

2. Wearable technology

- Definition of wearable technology
- Ask if they know of any wearable devices and demonstration of commercial activity devices
- Ask if they like this kind of technology and if so why:
- Would you use it?
- How often will you be willing to wear it? Daily?
- Ask what they do not like about this kind of technology and if so, why:
- What would put you off using such technology?

3. Feelings about wearable medical technology

- How are you doing in general in dealing with your disease?
- Do you think wearable technology would help your current situation? If so, how?
- Can you think of any disadvantages of this sort of wearable technology?
- How do you view this technology in comparison to conventional forms of treatment? And why?
- Do you see yourself using this kind of technology? If so, how?

4. Impact on relationships

- If you did decide to use this technology, how do you think it would impact on your daily interactions with others?
- Do you think it would change how you interact with medical professionals?
- Do you think it would affect your home life/working environment (if applicable)?
- What are your views on data privacy? Would you be worried about data privacy when using a wearable device?

5. Closing

- Is there anything else you would like to say about what we have discussed?
- Thank everyone for their time and useful participation
