# Supplementary figures and images for: Monitoring and Managing Lifestyle Behaviors Using Wearable Activity Trackers: Mixed Methods Study of Views From the Huntington Disease Community
Source: JMIR Form Res. 2022 Jun 29;6(6):e36870. doi: 10.2196/36870 (PMC9280464; doi:10.2196/36870)

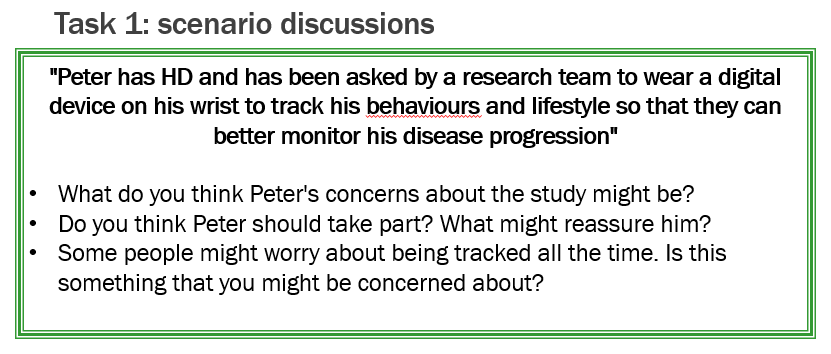

Supplement: Multimedia Appendix 3 [file formative_v6i6e36870_app3.docx]
